# First Pediatric Case of Clinically-Diagnosed Penicillin G–Induced Hemorrhagic Cystitis

**DOI:** 10.7759/cureus.32246

**Published:** 2022-12-06

**Authors:** Ryo Hayashi, Yasuko Urushihara, Hirotaka Ishido, Yoichi Iwamoto, Seigo Korematsu, Satoshi Masutani

**Affiliations:** 1 Pediatrics, Saitama Medical Center, Saitama Medical University, Kawagoe, JPN

**Keywords:** urinary bladder, adverse effect, infective endocarditis, hematuria, penicillin-g, hemorrhagic cystitis

## Abstract

Hemorrhagic cystitis is a diffuse inflammatory disease of the urinary bladder associated with macrohematuria. Several cases of hemorrhagic cystitis caused by penicillin G have been reported in adults but not children. Here we describe the first pediatric case of clinically-diagnosed penicillin G-induced hemorrhagic cystitis. The patient was a 9-year-old boy with a ventricular septal defect, chromosomal abnormalities, and infective endocarditis caused by *Abiotrophia defectiva*. After approximately four weeks of penicillin G administration, he had a culture-negative major hemorrhage with a clot. The hematuria resolved one week after penicillin G discontinuation, and a drug lymphocyte stimulation test for penicillin G was positive. In conclusion, penicillin G can also induce hemorrhagic cystitis in children. When large doses of penicillin G are used for long periods in adults or children, the patient should be monitored for hemorrhagic cystitis.

## Introduction

Hemorrhagic cystitis (HC) is a diffuse inflammatory condition of the urinary bladder with an infectious or non-infectious etiology that results in macrohematuria due to bleeding from the bladder mucosa [1]. HC is mainly caused by viral infections, radiation, allergic reactions, and chemical agents. As HC can be serious and may require invasive diagnostic evaluations, recognizing the possible causes is of clinical importance. Penicillin G (PCG) is an antibiotic to treat various bacterial infections, including pneumonia, sepsis, and endocarditis, caused by bacteria such as staphylococci and streptococci [2].To our knowledge, HC induced by PCG has rarely been reported in adults [3] but not in children. Herein, we report a clinically-diagnosed pediatric case of PCG-induced HC.

## Case presentation

A 9-year-old boy was transferred to our center to continue and optimize treatment for infective endocarditis (IE) on the 22nd febrile day. He was born at 37 weeks and 0 days gestation with a birth weight of 1543 g. A chromosomal abnormality (47, XY,+mar in 3 cells /46, XY in 27 cells) was previously diagnosed. The patient was followed up at the outpatient clinic for short stature requiring growth hormone injections, a ventricular septal defect, and a bicuspid aortic valve. Sixteen days before the fever developed, a tooth was extracted without antibiotic prophylaxis. Although no vegetation was detected by transthoracic echocardiography at the previous hospital, the diagnosis of IE [4] was made due to three blood cultures positive for Abiotrophia defectiva, high fever, congenital heart defect, serological evidence of active infection consistent with IE, and the present history. Vancomycin (VCM) and gentamicin (GM) were administered, and the fever soon resolved.

**Figure 1:**
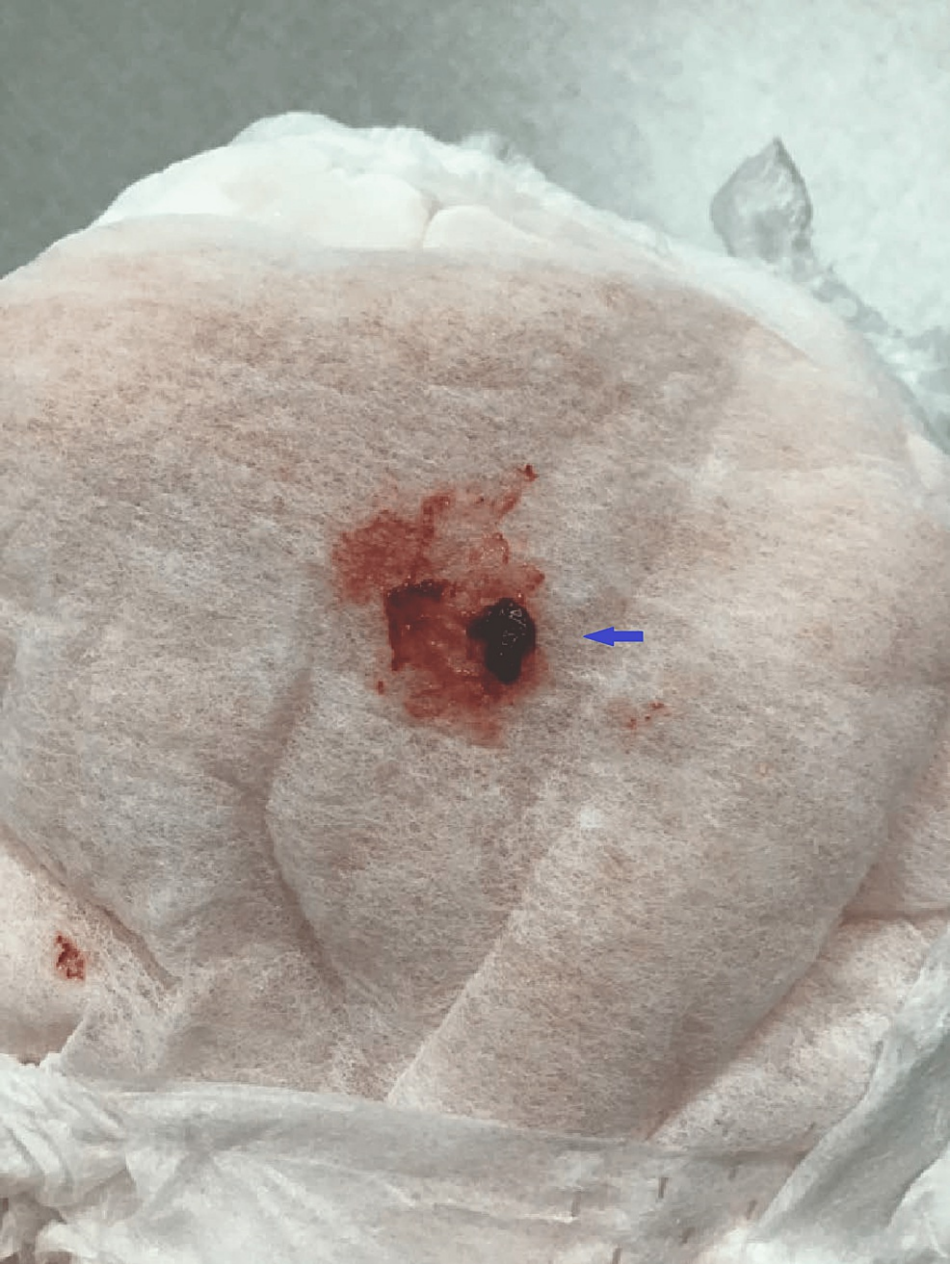
Gross findings of hematuria. A clot is visible in the gross hematuria in the patient’s diaper.

 In our center, we switched the VCM to PCG (1 million U every 6hr) and continued the GM (13mg every 8hr) while his body weight was 15.8 kg. Further blood cultures were negative, and we decided to administer the PCG for six weeks with GM according to the Japanese guidelines for the prevention and treatment of IE (JCS, 2017) [5]. Just before the end of the administration period, at approximately five weeks of PCG and eight weeks of GM treatment, it was revealed that he had macrohematuria for one week. He reported no abdominal pain, but he did experience frequent urination and nocturnal urinary incontinence. The macrohematuria was accompanied by a clot (Fig 1).

Laboratory data are shown in the Table 1. The uniform urinary red blood cell morphology and the absence of a urinary column indicated that the hematuria was not from the kidneys but from the bladder. Other data did not suggest streptococcal or hepatitis B infection and systemic erythematosus. Ultrasonography showed unremarkable results, a negative urinary culture, and pathological cytopathology revealed eosinophils in addition to neutrophils.

**Table 1:** Laboratory data. AST: Aspartate aminotransferase; ALT: Alanine aminotransferase; LD: Lactate dehydrogenase; g-GTP: g-Glutamyl transpeptidase; HPF: High power field; Na: Sodium; Cl: Chlorine; K: Potassium; CRP: C-reactive protein

Blood chemistry		Complete blood count		Urinalysis	
Total protein	6.2 g/dL	White blood cell	7100 /mL	Color	Orange
Albumin	3.6 g/dL	Red blood cell	369*10^4^ /mL	Specific gravity	1.014
AST	20 U/L	Hemoglobin	11.0 g/dL	pH	6.5
ALT	11 U/L	Hematocrit	32.3%	Nitrite reaction	-
LD	243 U/L	Platelet	235*1000/mL	Protein	+3
g-GTP	14 U/L			Glucose	±
Creatinine	0.38 mg/dL			Ketone	-
Urea nitrogen	3.5 mg/dL			Urobilinogen	Normal
Na	138 mEq/L			Occult blood	+3
Cl	101 mEq/L			Red blood cell	> 100/HPF
K	4.4 mEq/L			Dysmorphic red blood cell	<10%
Total bilirubin	0.4 mg/dL			White blood cell	> 100/HPF
C-reactive protein	0.26 mg/dL			Cast	(-)

The hematuria and proteinuria rapidly ameliorated and disappeared by one week after discontinuation of the PCG (total, six weeks) and GM (total, nine weeks). Three months after the HC diagnosis, a drug lymphocyte stimulation test (DLST) for PCG was positive as a stimulation index of 3.4 [6], while that for GM was negative. We eventually clinically diagnosed PCG-induced HC by these findings and his clinical course. The HC has not recurred, and the patient is awaiting surgery for the ventricular septal defect.

## Discussion

To our knowledge, this is the first pediatric case of PCG-induced HC. PCG, a classical antibiotic, still plays an important therapeutic role in specific conditions such as IE. As such states require therapy with a large amount and long duration of PCG, possible drug-induced side effects need careful monitoring. Three previously reported adult HC cases induced by PCG [3,6-8] involved IE treated with aminoglycoside [6-8]. In those situations that require a large amount of PCG for a long duration, a routine urinary qualitative test may be helpful, especially in pediatric cases, to minimize the delay of noticing the onset of HC, as in this case.

Several interventions, with varying degrees of effectiveness, are currently used in managing radiation- or chemotherapy-induced refractory HC. Typically, manual irrigation of the bladder with a urethral catheter and continuous bladder irrigation is used. Treatment of refractory HC includes several oral and intravesical medications, oxygen therapy, and surgical options [9]. Fortunately, the HC in the current pediatric case resolved following the discontinuation of PCG after the IE treatment period without needing these interventions.

In this case, the time to HC recovery was relatively rapid when we considered that 1-3 weeks were required for PCG-induced HC in an adult [3]. Although the precise mechanism of penicillin-induced HC is not known, a penicillin-related immune reaction may damage the bladder mucosa [3], or penicillin itself or metabolites may be directly toxic to the bladder. Because most patients develop symptoms after prolonged exposure to penicillin, direct bladder irritation is possible. However, there is more evidence supporting an immunological basis. The entity is well-known yet rare and has been recognized for a long. This immunological basis mechanism is supported by findings such as peripheral eosinophilia or eosinophilic infiltration into the bladder mucosa [3], a positive DLST result for PCG [6], and immunofluorescence microscopic findings of an intense immunoglobulin G and M and faint third component of complement deposition in the submucosa in a case of methicillin-induced hematuria [10]. Although we were unable to directly assess the bladder mucosa because his hematuria disappeared shortly after the cessation of PCG, a limitation of this case report, the clinical diagnosis of PCG-induced HC in our case was made according to the patient’s clinical course [3] and confirmed by the positive DLST result for PCG [6] and negative DLST result for GM. The results of DLST show the presence of PCG-specific T cells, providing evidence of HC due to a type 4 allergic reaction. DLST is often examined in a suspected drug and newborn milk allergy patients. This case’s clinical course suggests that PCG induced HC through type 4 allergic reaction, and DLST is also helpful in examining the cause of HC.

In this case, a tooth was extracted without antibiotic prophylaxis with a ventricular septal defect and a bicuspid aortic valve, and later IE occurred. The American heart association guidelines 2007 [11] significantly reduced the indications for prophylactic oral antimicrobial therapy only to the highest-risk group. The lack of prophylactic oral antimicrobial treatment in this patient not having the highest risk met that guideline [11]. In a recent analysis, however, IE due to viridans group streptococci showed a significant increasing trend in those aged 10 years and older when compared before and after that guideline [12]. Prophylactic antimicrobial therapy in the moderate-risk group patients is weakly recommended in the Japanese guidelines for IE 2017 [5]. Further prospective studies are needed to clarify better the indications for prophylactic administration in the moderate risk group. This case has a minor chromosomal abnormality, but no reports show an association with susceptibility to infection.

In summary, this is the first case report of a child with clinically-diagnosed HC caused by long-term PCG use, while a few cases have been reported in adults. Immediate improvement after PCG discontinuation and a positive DLST test helped diagnose PCG-induced HC.

## Conclusions

PCG is a classic antibiotic, but it still plays an important therapeutic role and is used in clinical practice. PCG can also induce HC in children. Recognizing HC as an adverse reaction to PCG may enable the early withdrawal of PCG as a causative agent and early recovery, avoiding unnecessary invasive examinations. Thus, it should be noted by clinicians who treat pediatric populations that PCG can also induce HC in children. In addition, DLST helps examine the cause of HC.
